# Using a probabilistic approach to derive a two-phase model of flow-induced cell migration

**DOI:** 10.1016/j.bpj.2024.02.017

**Published:** 2024-02-28

**Authors:** Yaron Ben-Ami, Joe M. Pitt-Francis, Philip K. Maini, Helen M. Byrne

**Affiliations:** 1 Wolfson Centre for Mathematical Biology, Mathematical Institute, University of Oxford, Oxford, UK; 2 Department of Computer Science, University of Oxford, Oxford, UK; 3 Ludwig Institute for Cancer Research, University of Oxford, Oxford, UK

## Abstract

Interstitial fluid flow is a feature of many solid tumors. In vitro experiments have shown that such fluid flow can direct tumor cell movement upstream or downstream depending on the balance between the competing mechanisms of tensotaxis (cell migration up stress gradients) and autologous chemotaxis (downstream cell movement in response to flow-induced gradients of self-secreted chemoattractants). In this work we develop a probabilistic-continuum, two-phase model for cell migration in response to interstitial flow. We use a kinetic description for the cell velocity probability density function, and model the flow-dependent mechanical and chemical stimuli as forcing terms that bias cell migration upstream and downstream. Using velocity-space averaging, we reformulate the model as a system of continuum equations for the spatiotemporal evolution of the cell volume fraction and flux in response to forcing terms that depend on the local direction and magnitude of the mechanochemical cues. We specialize our model to describe a one-dimensional cell layer subject to fluid flow. Using a combination of numerical simulations and asymptotic analysis, we delineate the parameter regime where transitions from downstream to upstream cell migration occur. As has been observed experimentally, the model predicts downstream-oriented chemotactic migration at low cell volume fractions, and upstream-oriented tensotactic migration at larger volume fractions. We show that the locus of the critical volume fraction, at which the system transitions from downstream to upstream migration, is dominated by the ratio of the rate of chemokine secretion and advection. Our model also predicts that, because the tensotactic stimulus depends strongly on the cell volume fraction, upstream, tensotaxis-dominated migration occurs only transiently when the cells are initially seeded, and transitions to downstream, chemotaxis-dominated migration occur at later times due to the dispersive effect of cell diffusion.

## Significance

It is well known that interstitial flow biases the direction of cell migration, and that this phenomenon has important implications for tumor metastasis. Here, using a probabilistic approach, we develop a two-phase model for the directed migration of cells under the mechanochemical stimuli induced by fluid flow. Previous experimental findings have suggested the presence of competing tensotaxis and chemotaxis stimuli, resulting in transitions in the dominant mode of migration as the flow conditions and cell density are varied. The current model enables us to examine the competing signal hypothesis, to generate predictions about how the balance between these competing mechanisms changes over time and, thus, to determine the conditions under which a transition from upstream to downstream cell migration occurs.

## Introduction

Cells can sense a variety of chemical and mechanical cues that may bias their movement. In healthy tissues, cells migrate in response to multiple environmental cues: examples include morphogenesis, wound healing, and the stimulation of an immune response to infection (1). At the same time, many diseases are characterized by excessive (or insufficient) directed cell migration: examples include tumor invasion and metastasis to adjacent tissues (2,3) and impaired wound healing caused by diabetes (4).

Fluid flow has been found to promote tumor cell migration in several different ways (3,5,6,7). Interstitial fluid flow in solid tumors is known to be higher than in healthy tissues due to growth-induced increases in interstitial pressure and leaky blood vessels. Consequently, interstitial flow has been suggested as a contributor to cell migration and metastasis (8,9).

In vitro experiments (5) have shown that fluid flow may impact the directed movement of cells in several different ways. On the one hand, extracellular fluid flow increases the pressure on the upstream part of the cell and, consequently, the cell increases the adhesion forces it exerts on the extracellular matrix (ECM) in this region. In turn, the localized tension at the front of the cell leads to actin localization and protrusion in this region, contributing to migration against the direction of flow (6). This mechanism, which is dominant in three-dimensional (3D) cell cultures, is similar to the mechanism underlying *durotaxis*, where cells on a 2D substrate migrate in response to gradients in the mechanical stiffness of the substrate (10,11). Cell movement in response to gradients in cell-ECM adhesion forces has been termed *rheotaxis* in (6), but here we refer to it as *tensotaxis* (12) to emphasize the role of fluid-induced stress (rather than velocity gradients) on this type of movement. In addition to upstream directed movement induced by tensotaxis, *autologous chemotaxis* drives cell movement downstream. Here, the flow advects cell-secreted ligands, creating transcellular gradients of chemokines. The ligands bind to specific receptors on the cell surface, inducing cell polarization in the direction of higher chemokine concentrations and driving downstream, chemotactic migration. This autologous signaling mechanism has been observed by Shields et al. (3), where tumor cells have been shown to migrate downstream by binding self-secreted CCL21 ligands to the CCR7 receptors.

**Figure 1:**
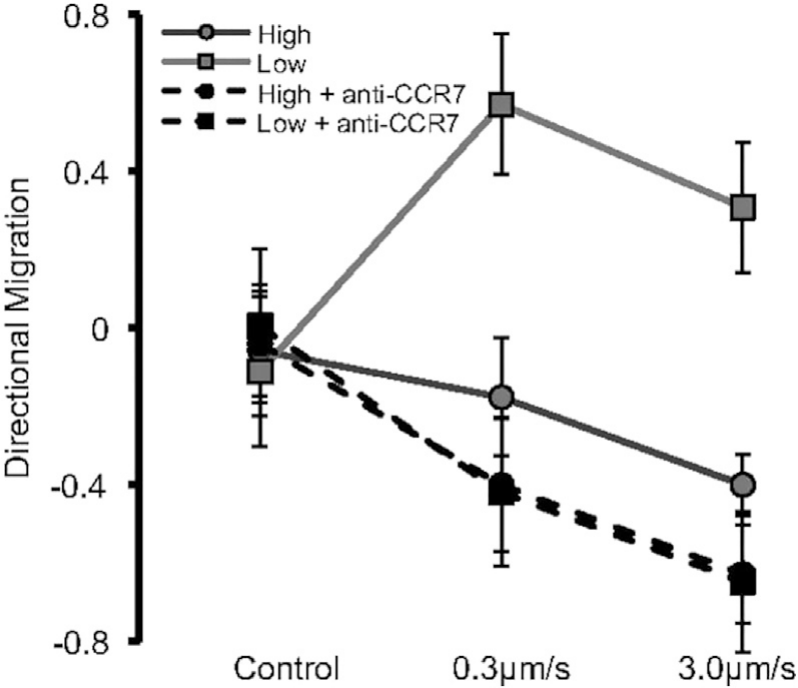
Experimental results, reproduced with permission from (5), showing the directional migration score (positive [negative]––most cells travel downstream [upstream], see (5) for details) as a function of the strength of interstitial flow and for different cell seeding densities (“high” and “low” refer to seeding densities of $25\times 10^{4}$ and $5\times 10^{4}$ cells/mL, respectively). The dashed lines show that upstream migration prevails when the CCR7 receptor signaling pathway is blocked, interrupting the downstream-oriented autologous chemotaxis.

In experiments by Polacheck et al. (5), cancer cells were seeded in a microfluidic channel and subject to fluid flow. The distribution of cell velocities was measured and the average migration direction (with respect to the flow direction) was evaluated; the local flow direction experienced by the cells was evaluated by numerically simulating the flow field in the microfluidic device. The results, reproduced in Fig. 1, show that the dominant mode of migration switched between downstream (with the flow direction; positive values of directional migration in Fig. 1) and upstream (against the flow direction; negative values of directional migration in Fig. 1) as the cell density increased. However, when the CCR7 receptor signaling pathway was blocked, upstream migration was found to prevail regardless of the cell density, supporting the observations by Shields et al. (3) regarding CCR7-dependent, downstream-oriented autologous chemotaxis. In addition, for all of the experimental curves shown in Fig. 1, an increase in the interstitial flow led to a higher tendency of the cells to migrate upstream. These results motivate the question of how different properties of cells, and the mechanochemical landscape they sense, affect their migration directions. In this paper, we show how mathematical modeling can shed light on the mechanisms regulating the direction of collective cell migration in a flow as system parameters vary.

Models of chemotactic migration go back to the highly influential work of Keller and Segel (13). More recently, chemotaxis has been considered in the context of two-phase cellular tissue models (14,15) by formulating mass and momentum balance of the cell and fluid phases coupled to the transport equation for chemoattractant propagation in the fluid phase. The ability of multiphase models to incorporate coupled interactions between cells, fluid, and chemoattractants makes them a natural framework for describing the mechanisms involved in mechanochemical transduction of cells subject to interstitial fluid flow.

While models for chemotaxis are prevalent (see the extensive review in (16)), models for tensotaxis are less common. In a recent related work (17), a generalized Keller-Segel model was applied to study the combined effect of rheotaxis (directed movement in response to flow velocity field) and chemotaxis on the aggregation of swimming organisms. However, in (17) a single-phase model was formulated; thus, the coupled interactions of the organisms and fluid were not considered. Evje and co-workers (18,19) have formulated a multiphase model that combined the competing mechanisms of downstream-oriented autologous chemotaxis and upstream force, introduced ad hoc by inverting the direction of fluid drag force acting on the cells. Their model have been successful in reproducing the transition between downstream and upstream migration as the cells’ volume fraction increases, as was observed in (5). However, the heuristic assumption of inverting the direction of drag force does not explain the mechanisms underlying this type of migration and does not allow any generalization of the model to more complicated scenarios where different sources of mechanical stimulus exist. More recently, Rosalem et al. (12) derived a single-phase model for the tensotactic migration of cells, where the cell flux was assumed to be proportional to the transcellular pressure gradient. They verified that, in the presence of flow, this mechanism leads to upstream migration of cells; however, they did not consider the opposing effect of chemotactic migration. Consequently, it remains to be established what parameters (other than cell volume fraction) affect the direction of cell migration and what parameter regimes support downstream, rather than upstream, cell migration.

An additional drawback of existing macroscopic multiphase models for cell migration (e.g., (14,18)) is that directed migration is modeled as an internal force exerted by the cells on their self (source terms in the cell momentum balance). Therefore, the cell speed increases with the strength of the stimulus. This contradicts some experimental findings, which show that individual cells bias their directionality in response to the external cues, but their speed of migration is not correlated with their directionality (5,11,20).

The goal of this work is to derive a two-phase model for cell migration subject to flow-induced mechanochemical stimuli. The chemotactic and tensotactic cues are viewed as external signals that bias the probability that a cell moves in a certain direction while the magnitude of its speed remains constant. This situation resembles the kinetic model developed by Hillen (21) to describe contact guidance of cell migration along the ECM fiber network. In that model, Hillen (21) used a transition probability function (TPF) to describe the velocity-jump process of the cells, biased by the structure of the ECM network. The use of a TPF was originally introduced in the pioneering work by Alt and co-workers (22) and has been used since to describe many aspects of directed cell migration (see the review by Perthame (23) and references cited therein). Building on these previous works, we propose a mesoscopic kinetic equation to describe the evolution of the cells’ probability density function in response to the external stimulus induced by fluid flow; here, the stimulus induces a cell velocity jump via a specifically tailored TPF. We then apply velocity-space averaging to derive continuum equations for the spatiotemporal evolution of the cells' volume fraction and flux in response to forcing terms depending on the local direction and magnitude of the mechanochemical stimulus. Using a combination of numerical simulations and asymptotic analysis, we delineate the parameter regimes for which cell migration transitions from downstream to upstream.

The remainder of the manuscript is structured as follows. In the methods we introduce our two-phase model for cell migration in response to flow-induced mechanochemical stimuli; we then introduce the 1D model problem that is the focus of this paper, and use asymptotic methods to derive the critical conditions for transition between downstream and upstream migration in the limit of small stimulus. In the results and discussion we present numerical results describing the spatiotemporal dynamics of the cell layer for different parameter regimes supporting different modes of migration; then, we compare predictions from the asymptotic analysis with numerical results derived from the full model regarding the conditions under which migration switches between downstream and upstream regimes. In the conclusion we summarize our findings and outline possible directions for future research.

## Methods

### Model formulation

In this section we introduce a model for cell migration in the presence of interstitial fluid flow, motivated by in vitro experiments that show that interstitial flow can induce mechanochemical stimuli biasing the direction of cell migration (3,5). In the present model we view the mechanochemical cues as external signals that regulate the probability that the cells move in a certain direction, and assume that the magnitude of the cell speed remains constant. In more detail, we seek to formulate a kinetic model for the probability density function, f(x,t,ξ), that the velocity of a cell in a neighborhood of spatial position x, at time *t*, has orientation vector ξ. In the following subsection we formulate a dimensional model in an arbitrary number of dimensions; in the subsequent subsection we consider a simplified 1D version of the model and then nondimensionalize the governing equations using the characteristic scales of the system which we introduce therein.

#### Probabilistic model for cell migration

We consider a mesoscopic kinetic model for the cells’ probability density function, f(x,t,ξ). We assume that the cells travel at a drift velocity $U_{c}\xi$, where the cell speed, $U_{c}$, is constant and ξ is a unit vector representing the cell velocity orientation, which evolves in response to the external stimulus sensed by the cells. This change in cell velocity orientation is modeled using a velocity-jump process induced by a TPF, F(x,t,ξ). We assume further that the cells perform an unbiased random walk (modeled as a microscopic space-jump process) superimposed on their directed movement. Accordingly, we model the change in the cells’ probability density function, f(x,t,ξ), using the following mesoscopic kinetic model:

(Equation 1) $$\frac{\partial f}{\partial t}+U_{c}\xi \cdot \nabla f=\frac{1}{\tau }\left[\int_{\xi^{\prime }\in V}F\left(x,t,\xi \right)f\left(x,t,\xi^{\prime }\right)d\xi^{\prime }-f\right]+D_{c}\nabla^{2}f\text{,}$$

where $D_{c}$ denotes diffusivity due to the unbiased random motion; ξ and $\xi^{\prime }$ mark the current and previous cell velocity orientation, respectively. The transition probability, *F*, represents the rate at which the orientation vector changes, and biases the probability density function in the direction of the stimulus; the constant, *τ*, represents the relaxation time over which the cell responds to the external signal. The integration in Eq. 1 is carried out over all cells at (x,t) (i.e., over all possible [previous] velocity orientations, $\xi^{\prime }\in V$, where V is the set of vectors pointing from the origin to the surface of a unit sphere). We assume that F=F(x,t,ξ) is only a function of the current cell velocity (after jump) and not a function of the previous velocity (before jump). Therefore, Eq. 1 can be written as

(Equation 2) $$\frac{\partial f}{\partial t}+U_{c}\xi \cdot \nabla f=\frac{1}{\tau }\left[\frac{\phi }{V_{c}}F-f\right]+D_{c}\nabla^{2}f\text{,}$$

where we assume that all cells have volume, $V_{c}$. Thus, we define the cell volume fraction as

$$\phi =V_{c}\int_{\xi \in V}f\left(x,t,\xi \right)d\xi \text{.}$$

We note that the kinetic formulation in Eq. 1 contains terms that arise from both space- and velocity-jump processes. Since this approach is nonstandard, we now include more explanatory details. The velocity-jump process induced by the transition probability, *F*, will introduce a diffusion term at the second moment of *f*. However, the cell “diffusivity” will then scale as $U_{c}^{2}\tau$ (see, e.g., (24)). Under this scaling, we estimate that, for tumor cell migration, the diffusion term will be subdominant to the drift term. We note that this may contradict some experimental results on tumor cell migration for which the diffusive displacement is typically larger than the directed displacement (e.g., (20,25)). In contrast to, for example, a run-and-tumble process that characterizes some bacteria, with cell migration there is no particular reason to assume a priori that the Brownian and directed motion of cells share similar speeds, particularly when the latter involves sensing, which may reduce its speed relative to the speed of random motion. By assuming two separate stochastic processes (i.e., an isotropic space-jump process and an anisotropic [stimulus-induced] velocity-jump process), we can control the scales of both diffusive and directed motion and, thus, consider their effects separately. Painter and Hillen (26) used a transition probability that includes both isotropic and anisotropic parts to model the two separate processes; however, we prefer to use an isotropic space-jump process because it allows for simpler closure of the macroscopic model.

In Eq. 1, cell-cell interactions and cell-volume exclusion are neglected. As such, the model is suitable to describe situations in which the cell volume fraction takes low to moderate values. While the diffusion term in Eq. 1 may mimic the effect of intercellular repulsion, other phenomena related to collective cell migration (27,28) are neglected in our model formulation to focus attention on the way in which flow-induced stimuli direct cell migration. In Eq. 1 we also neglect cell proliferation and death since we aim to model migration dynamics at much shorter time scales.

We define a stimulus vector, s=|s|η, where |s| represents its magnitude and η is a unit vector, such that *F* is maximized when the cell velocity and stimulus vector are aligned, and *F* decreases monotonically as the angle between ξ and η increases. A simple functional form that captures this behavior is given by

(Equation 3) F(s(x,t),ξ)=Aexp[s·(ξ−η)],

where the normalization factor *A* is chosen so that *F* satisfies

(Equation 4) $$\int_{\xi \in V}Fd\xi =1\text{,}$$

to ensure conservation of mass. Eq. 3 states that the velocity-jump process depends solely on the local stimulus vector.

We define θ∈[0,π] as the angle between ξ and η, such that the 3D integration dξ=sinθdθdφ, where φ∈[0,2π] is the polar angle in the plane perpendicular to η. Therefore, the integral in Eq. 4 reads

(Equation 5) $$\int_{\xi \in V}Fd\xi =2\pi A\int_{0}^{\pi }\;\exp\;\left[\left|s\right|\left(\cos\;\theta -1\right)\right]\sin\;\theta d\theta =2\pi A\frac{\left(1-e^{-2\left|s\right|}\right)}{\left|s\right|}\text{.}$$

Combining Eqs. 4 and 5 we have

(Equation 6) $$A=\frac{\left|s\right|}{2\pi \left(1-e^{-2\left|s\right|}\right)}\text{.}$$

Accordingly, *F* is given by

(Equation 7) $$F=\frac{\left|s\right|}{2\pi \left(1-e^{-2\left|s\right|}\right)}\;\exp\;\left[s\cdot \left(\xi -\eta \right)\right]\text{.}$$

We multiply Eq. 2 by $V_{c}$ and integrate over all possible cell velocity orientations, ξ∈V. Then, together with the normalization condition of *F* (Eq. 4), we have the macroscopic cell conservation equation

(Equation 8) $$\frac{\partial \phi }{\partial t}+\nabla \cdot \psi =D_{c}\nabla^{2}\phi \text{,}$$

where the cell flux, ψ, is given by

$$\psi =V_{c}U_{c}\int_{\xi \in V}\xi fd\xi \text{.}$$

To close the model we require an additional equation for ψ, and a natural choice is a momentum balance equation. To derive the momentum balance, we multiply Eq. 1 by $V_{c}U_{c}\xi$ and integrate over all possible velocity orientations to obtain

(Equation 9) $$\frac{\partial \psi }{\partial t}+U_{c}^{2}V_{c}\nabla \cdot \int_{\xi \in V}\xi \xi^{T}fd\xi =\frac{1}{\tau }\left(\phi U_{c}\int_{\xi \in V}F\xi d\xi -\psi \right)+D_{c}\nabla^{2}\psi \text{.}$$

The system of macroscopic Eqs. 8 and 9 is not closed due to the presence of the second-order moment in the left-hand side of Eq. 9. There is a wide body of literature concerning the formal derivation of macroscopic equations from kinetic models (see, e.g., (24) for closures of cell migration models and (29) for derivation of hydrodynamic equations from gas kinetic theory). However, for the present case of tumor cell migration, a simpler way to close the model is facilitated by the following dimensional analysis. We assume that the characteristic length scale of the system is *L* and the characteristic time scale is the relaxation time, *τ*. The characteristic scale of the cell flux is $\psi ∼U_{c}$. Applying this scaling to Eq. 9 we have

(Equation 10) $$\frac{\partial \tilde{\psi }}{\partial t}+\frac{U_{c}V_{c}\tau }{L}\tilde{\nabla }\cdot \int_{\xi \in V}\xi \xi^{T}fd\xi =\phi \int_{\xi \in V}F\xi d\xi -\tilde{\psi }+\frac{D_{c}\tau }{L^{2}}{\tilde{\nabla }}^{2}\tilde{\psi }\text{,}$$

where tildes denote nondimensional variables and operators. From Eq. 2, we estimate that the characteristic magnitude of *f* is $F\phi /V_{c}$ (this is the equilibrium probability in the limit of small cell speed and diffusivity). Assigning $f∼F\phi /V_{c}$ to the second-order moment in Eq. 10 we have

(Equation 11) $$V_{c}\int_{\xi \in V}\xi \xi^{T}fd\xi ∼\frac{\left|s\right|}{2\pi \left(1-e^{-2\left|s\right|}\right)}\int_{\xi \in V}\xi \xi^{T}\;\times \exp\;\left[s\cdot \left(\xi -\eta \right)\right]d\xi \leq O\left(1\right)\text{,}$$

where it can readily be shown that the absolute magnitude of the components of the tensor in Eq. 11 is bounded such that they lie in the interval [0,1] for all s. Therefore, we assume that

(Equation 12) $$\frac{U_{c}V_{c}\tau }{L}\tilde{\nabla }\cdot \int_{\xi \in V}\xi \xi^{T}fd\xi ∼O\left(\frac{U_{c}\tau }{L}\right)\text{.}$$

For tumor cell migration we estimate that the characteristic cell speed is $U_{c}∼10\;\mu m/h$ (5,20) and that the relaxation time is of the order of minutes to a few hours τ≲1h (30). In order for the macroscopic model to be valid, we assume that the characteristic length scale is much larger than the dimensions of a single cell, L≫10μm/h. These characteristic scales lead to the conclusion that

$$\frac{U_{c}\tau }{L}\ll 1$$

and, therefore, that the second-order moment term (Eq. 12) can generally be neglected in Eq. 9 in the context of tumor cell migration. We expect that a Chapman-Enskog expansion in the small parameter $U_{c}\tau /L$, similar to that carried out by Hillen (21), would result in $O\left(U_{c}\tau /L\right)$ diffusive correction terms. However, such an expansion is beyond the scope of the current article.

We note that, in this model, the length scale parameter, $U_{c}\tau$, represents the mean-free path of the cells: it describes the characteristic distance a cell covers before changing direction. Therefore, the assumption that the parameter $U_{c}\tau /L\ll 1$ means that, in the macroscopic limit, cells undergo many velocity jumps along the macroscopic length scale. This parameter emerges as the equivalent to the Knudsen number in the kinetic theory of gases (29), which represents the ratio of the molecular mean-free path to the macroscopic length scale. Similarly to our model, in the kinetic theory of gases the derivation of macroscopic hydrodynamic equations from the kinetic Boltzmann equation is facilitated in the limit of small Knudsen number.

While it is possible to continue with the nondimensional formulation given by Eq. 10, we now return to the dimensional formulation and postpone nondimensionalization to the section where we introduce the model-problem setup. Reverting to the dimensional formulation in Eq. 9 and evaluating the transition probability integral on the right-hand side, we have

(Equation 13) $$\int_{\xi \in V}F\xi d\xi =\frac{\left|s\right|}{2\pi \left(1-e^{-2\left|s\right|}\right)}\int_{\xi \in V}\xi \;\exp\;\left[s\cdot \left(\xi -\eta \right)\right]d\xi =\left(\coth\left(\left|s\right|\right)-{\left|s\right|}^{-1}\right)\eta \text{,}$$

where the components in directions perpendicular to η vanish due to symmetry.

Finally, neglecting the second-order moment term in Eq. 9 and substituting from Eq. 13 we have

(Equation 14) $$\frac{\partial \psi }{\partial t}=\frac{1}{\tau }\left[\phi U_{c}\left(\coth\left(\left|s\right|\right)-{\left|s\right|}^{-1}\right)\eta -\psi \right]+D_{c}\nabla^{2}\psi \text{.}$$

We note that the cell flux source term in Eq. 14 is in the direction of the stimulus and is monotonically increasing with the stimulus magnitude, |s| (vanishes as |s|→0 and attains a maximal value of $\phi U_{c}$ as |s|→∞). This source term acts as an effective “force” driving cells in the direction of the stimulus. In what follows, we propose a constitutive model for the stimulus, s, which depends on the local mechanochemical cues sensed by the cells.

#### Constitutive model for the mechanochemical stimulus

Cancer cells react to a variety of chemical and mechanical stimuli. We introduce a stimulus potential, Φ, such that

(Equation 15) $$s=-l_{c}\nabla \Phi \text{,}$$

where $l_{c}$ is a constant length, which should be at the scale of the cell length. We consider two stimulus potentials:

##### Chemotaxis

Binding of ligands to specific receptors on the membrane of cancer cells can polarize their movement in the direction of larger concentration of these ligands, leading to effective chemotactic migration (2). We model this process by assuming that the potential of the chemotactic stimulus is proportional to a chemokine concentration, *a*,

(Equation 16) $$\Phi_{C}=-\chi a\text{,}$$

where the constant *χ* represents the chemotactic potential per unit concentration.

##### Tensotaxis

Cells respond to local stress by biasing their movement in the direction of larger tension in their cell-ECM connections (5,6). When cells embedded in a 3D matrix are subject to interstitial flow, the cell response is usually stimulated by increased fluid pressure at the upstream part (the part facing the flow) of the cell, which causes the cell to generate tensile ECM adhesion forces in this region (and compressive forces in the downstream region) resisting the flow-induced drag force. In turn, the localized tension at the upstream part of the cell polarizes its movement in the direction of larger pressure (6). For simplicity, we model this process by viewing the tensotactic stimulus experienced by the cells as a potential that is proportional to the stresses acting on the cell in the direction normal to the cell's outer surface:

(Equation 17) $$\Phi_{T}=-\varpi \sigma_{nn}^{\text{ext}}\text{.}$$

In Eq. 17, *ϖ* represents the strength of the tensotactic potential per unit stress, and $\sigma_{nn}^{\text{ext}}\equiv n^{T}\sigma^{\text{ext}}n$ is the extracellular stress acting on the cell in the direction normal to its outer surface, where $\sigma^{\text{ext}}$ is the extracellular stress tensor and n is a unit vector normal to the cell surface. We note here that, in more general cases, the cells may be subject to other external stresses, such as shear stresses (31) that stimulate tensotaxis. Typically, when cells are embedded in a 3D matrix and subject to fluid flow, the dominant stress they experience is due to fluid pressure (5). Therefore, in this work we assume that Eq. 17 can be simplified to read

(Equation 18) $$\Phi_{T}=-\varpi p\text{,}$$

where *p* is the interstitial fluid pressure.

Finally, we write the total potential, Φ, as the sum of the chemotactic and tensotactic potentials, so that $\Phi =\Phi_{C}+\Phi_{T}$. Then, Eq. 15 becomes

(Equation 19) $$s=l_{c}\varpi \nabla p+l_{c}\chi \nabla a\text{.}$$

To close the model we introduce equations for *p* and *a*. In what follows we formulate the governing equations of the flow dynamics in the two-phase cell-fluid mixture, such that the fluid pressure and the concentration of the flow-advected chemokine can be evaluated.

#### Interstitial flow dynamics

We make a no-voids assumption for the cell-fluid mixture such that the volume fraction of the fluid phase is given by 1−ϕ. Then, the mass conservation equation of the fluid phase can be written as

(Equation 20) $$\frac{\partial \left(1-\phi \right)}{\partial t}+\nabla \cdot \left[\left(1-\phi \right)u_{f}\right]=0\text{,}$$

where $u_{f}$ is the fluid velocity. Combining Eq. 20 with Eq. 8 we have

(Equation 21) $$\nabla \cdot \left[\psi +\left(1-\phi \right)u_{f}\right]=D_{c}\nabla^{2}\phi \text{.}$$

Assuming the fluid flux is much larger than the cell flux, as is usual for biological tissues,

(Equation 22) $$\psi -D_{c}\nabla \phi \ll \left(1-\phi \right)u_{f}\text{,}$$

we can simplify Eq. 21 to

(Equation 23) $$\nabla \cdot \left[\left(1-\phi \right)u_{f}\right]=0\text{.}$$

We proceed by assuming that the momentum equation of the fluid phase involves a balance between the drag force exerted by the cells and the pressure gradient (for simplicity, we neglect intraphase viscous stresses). This balance can be written as a Darcy-type equation

(Equation 24) $$u_{f}-u_{c}^{\text{avg}}=-k_{H}g\left(\phi \right)\nabla p\text{,}$$

where $u_{c}^{\text{avg}}=\psi /\phi$ is the average cell velocity, and $k_{H}$ represents the hydrodynamic conductivity (permeability divided by viscosity). In Eq. 24, g(ϕ) describes how the drag depends on the cell volume fraction, *ϕ*. For simplicity, we use the popular Carman-Kozney relation (32) so that

(Equation 25) $$g\left(\phi \right)=\frac{{\left(1-\phi \right)}^{3}}{\phi^{2}}\text{.}$$

We chose the Carman-Kozney model since it is arguably the simplest isotropic model for hydraulic permeability that includes the effect of cell volume fraction. The dependence on the volume fraction is important as it ensures that the tensotactic stimulus increases as the cell volume fraction increases, as has been observed experimentally (5).

In Eq. 24 we can neglect the cell velocity with respect to the fluid velocity to obtain

(Equation 26) $$u_{f}=-k_{H}g\left(\phi \right)\nabla p\text{.}$$

Substituting from Eqs. 25 and 26 into Eq. 19, we can write the equation for the stimulus vector as

(Equation 27) $$s=-\frac{l_{c}\varpi }{k_{H}}\frac{\phi^{2}}{{\left(1-\phi \right)}^{3}}u_{f}+l_{c}\chi \nabla a\text{.}$$

Finally, we model the evolution of the chemokine concentration, *a*, using a reaction-advection-diffusion equation. We assume that the chemokine is secreted by the cells at a constant rate, $\beta_{p}$, and that $\beta_{d}$ is the rate (per unit concentration) at which it binds to receptors on the surface of the cells. Under these assumptions we obtain the following equation for the chemokine concentration

(Equation 28) $$\frac{\partial a}{\partial t}+\nabla \cdot \left[a\left(1-\phi \right)u_{f}\right]=\frac{\beta_{p}}{V_{c}}\phi -\frac{\beta_{d}}{V_{c}}\phi a+D\nabla^{2}a\text{.}$$

where *D* is the diffusion coefficient of the chemokine in the interstitial fluid.

Taken together, Eqs. 8, 14, 23, 27 and 28 form a closed system for the cell volume fraction, *ϕ*, and flux, ψ, the cell stimulus, s, fluid velocity, $u_{f}$, and chemokine concentration, *a*.

### Model problem: Cell layer subject to 1D flow

#### Formulation of a nondimensional 1D model

In this subsection we reduce the model developed in the previous subsection to a 1D model that describes the migration of a population of cells (initially localized around a particular spatial position) in a long microfluidic channel (i.e., we neglect cell fluxes into, or out of, the channel edges). This model will be used to provide a simple explanation of how and why the migration patterns of tumor cells change in response to changes in flow velocity and cell volume fraction observed by Polacheck et al. (5). While the geometry of the microfluidic device in (5) is not exactly identical to a long channel, the fluid velocity in the cell region of the experiments was primarily oriented in a single direction, along the axis of the channel. We thus view our 1D model as a reasonable approximation of the experimental setup. We consider a long channel that is aligned with the *x* axis, in which an initial cell layer is distributed normally around x=0 such that

(Equation 29) $$\phi \left(x^{*},t^{*}=0\right)=\bar{\phi }\;\exp\;\left[-{\left(\frac{x^{*}}{L^{*}}\right)}^{2}\right]\text{.}$$

**Figure 2:**
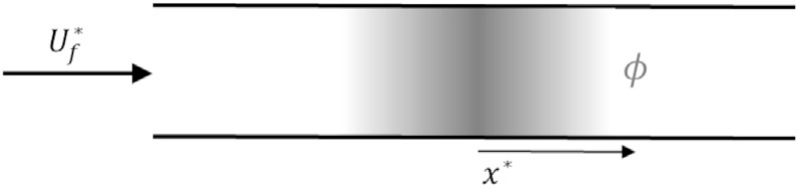
Schematic illustration of the 1D model problem.

In Eq. 29 and henceforth, we use asterisks to denote dimensional parameters, and the constants $\bar{\phi }$ and $L^{*}$ represent typical values of the cells’ initial volume fraction and layer size, respectively. The cells are subject to fluid flow, where in the far field as $\left|x^{*}\right|\gg L^{*}$ (i.e., in regions sufficiently far from the cell layer), the fluid velocity magnitude is $U_{f}^{*}$. A schematic of the 1D model problem is illustrated in Fig. 2.

We now simplify the equations derived in the model formulation subsection to 1D Cartesian geometry form and nondimensionalize them using the following scaling: we normalize length by the characteristic length of the initial distribution of cells, $L^{*}$; we scale the velocity by the far-field fluid velocity, $U_{f}^{*}$; accordingly, time is normalized by $L^{*}/U_{f}^{*}$. The chemokine concentration is scaled by its maximal equilibrium concentration, $a_{\text{eq}}^{*}=\beta_{p}^{*}/\beta_{d}^{*}$. The full set of independent and dependent nondimensional variables are given by

(Equation 30) $$x=\frac{x^{*}}{L^{*}},\;t=\frac{t^{*}U_{f}^{*}}{L^{*}},\;\phi ,\;\psi =\frac{\psi^{*}}{U_{f}^{*}},\;u_{f}=\frac{u_{f}^{*}}{U_{f}^{*}},\;\text{and}\;a=\frac{a^{*}}{a_{\text{eq}}^{*}}\text{.}$$

Then, Eq. 8 in a 1D nondimensional form reads

(Equation 31) $$\frac{\partial \phi }{\partial t}+\frac{\partial \psi }{\partial x}=\frac{1}{{\text{Pe}}_{c}}\frac{\partial^{2}\phi }{\partial x^{2}}\text{,}$$

where *ψ* is the *x*-component of ψ and

$${\text{Pe}}_{c}=\frac{U_{f}^{*}L^{*}}{D_{c}^{*}}\text{.}$$

Using physiologically relevant parameters we have $U_{f}^{*}∼1\;\mu m/s$ (5), $L^{*}∼100\;\mu m$, and $D_{c}^{*}∼1000\;\mu m^{2}/h$ (33), such that the interstitial fluid velocity is much larger than the diffusive velocity of cells, i.e., ${\text{Pe}}_{c}\gg 1$. We expect, however, that the cell flux will also be small, ψ≪1, such that we cannot neglect diffusive effects.

In one dimension it is easier to assume that the direction of the stimulus is constant, $\eta =\hat{x}$, where $\hat{x}$ is the unit vector in the positive *x*-direction. Then, the stimulus vector is given by

$$s=s\hat{x}\text{,}$$

where s∈(−∞,∞) is the *x*-component of the stimulus vector. We note that the stimulus vector is aligned with the *x* axis because, within the 1D model, the only gradients of the macroscopic fields are in the *x*-direction. However, the microscopic velocity, ξ, is still a 3D vector, in accordance with its definition in Eq. 1 et seq.

Reducing Eq. 14 to 1D and nondimensionalizing we have

(Equation 32) $$\frac{\partial \psi }{\partial t}=\frac{U}{T}\left[\coth\left(s\right)-s^{-1}\right]\phi -\frac{\psi }{T}+\frac{1}{{\text{Pe}}_{c}}\frac{\partial^{2}\psi }{\partial x^{2}}\text{,}$$

where

$$T=\frac{\tau^{*}U_{f}^{*}}{L^{*}}\;\text{and}\;U=\frac{U_{c}^{*}}{U_{f}^{*}}\text{.}$$

In Eq. 32 we made use of the antisymmetry of the source term in Eq. 14:

(Equation 33) $$\coth\left(s\right)-s^{-1}=\left\{ \begin{array}{cc} -\left(\coth\left(\left|s\right|\right)-{\left|s\right|}^{-1}\right), & s\leq 0\\ \;\coth\left(\left|s\right|\right)-{\left|s\right|}^{-1}, & s\geq 0. \end{array}\right.$$

Since the cell velocity is much smaller than the fluid velocity we will assume U≪1. The characteristic response time of cells, $\tau^{*}$, is in the range of minutes to hours (30) such that, based on the characteristic scales of length and fluid velocity introduced above, we can estimate that T∼10−100.

To evaluate the stimulus, *s*, we must solve for the fluid velocity and chemokine gradient. Starting from the fluid velocity, we consider the nondimensional 1D form of the cell-fluid mixture mass conservation Eq. 23

(Equation 34) $$\frac{\partial }{\partial x}\left[\left(1-\phi \right)u_{f}\right]=0\text{.}$$

Integrating Eq. 34 with respect to *x* we have

(Equation 35) $$u_{f}=\frac{1}{1-\phi }\text{.}$$

where we have assumed that $u_{f}{|}_{x\to -\infty }=1$, in accordance with the dimensional boundary condition, $u_{f}^{*}{|}_{x^{*}\to -\infty }=U_{f}^{*}$.

The chemokine transport equation in a 1D nondimensional form then reads

(Equation 36) $$\frac{\partial a}{\partial t}+\frac{\partial a}{\partial x}=\text{Da}\;\phi \left(1-a\right)+\frac{1}{\text{Pe}}\frac{\partial^{2}a}{\partial x^{2}}\text{,}$$

where

(Equation 37) $$\text{Da}=\frac{L^{*}\beta_{d}^{*}}{U_{f}^{*}V_{c}^{*}}\;\text{and}\;\text{Pe}=\frac{U_{f}^{*}L^{*}}{D^{*}}$$

are the chemoattractant Damkohler and Peclet numbers, respectively. Using the physiologically relevant parameter values of $U_{f}^{*}$ and $L^{*}$ introduced above, together with characteristic diffusivity of chemokines $D^{*}∼100\;\mu m^{2}/s$ (34,35), we can estimate that Pe∼1, meaning that diffusive effects are likely to be important. In the context of the present 1D model, the diffusive terms act to smooth the chemokine gradient and, thereby, to reduce the magnitude of the chemotactic cue in the center of the cell layer (with subdominant contributions at the edges of the domain). However, since $\text{Pe}\ll {\text{Pe}}_{c}$, we would need a very large domain to simulate, on the one hand, sufficiently large times to allow for cell migration while, on the other hand, avoiding boundary interactions of the chemokine at the channel edges. Therefore, to simplify the numerical calculations, we choose to neglect the diffusive term in Eq. 36 and view the results as the purely advective limit, bearing in mind that including diffusion would result in somewhat weaker chemotactic migration.

In addition, the time-derivative of the chemokine is associated with changes in the cell volume fraction such that

(Equation 38) $$\frac{\partial a}{\partial t}∼\text{Da}\frac{\partial \phi }{\partial t}∼O\left(\text{Da}\;U,\frac{\text{Da}}{{\text{Pe}}_{c}}\right)\ll 1\text{.}$$

Therefore, we can assume that the chemokine distribution is quasisteady, i.e., changes in the cell volume fraction lead to instantaneous adaption of the chemokine distribution. Under these assumptions and together with the vanishing of the chemokine at the channel inlet, $a{|}_{x\to -\infty }=0$, we can simplify Eq. 36 to

(Equation 39) $$a\left(x,t\right)=1-\;\exp\;\left(-\text{Da}\int_{-\infty }^{x}\phi \left(z,t\right)dz\right)\text{.}$$

Substituting from Eqs. 35 and 39 into the 1D form of Eq. 27 we have

(Equation 40) $$s=-K\frac{\phi^{2}}{{\left(1-\phi \right)}^{4}}+M\text{Da}\;\phi \;\exp\;\left(-\text{Da}\int_{-\infty }^{x}\phi \left(z,t\right)dz\right)\text{,}$$

where the nondimensional parameters

$$K=\frac{l_{c}^{*}\varpi^{*}U_{f}^{*}}{k_{H}^{*}}\;\text{and}\;M=\frac{l_{c}^{*}\chi^{*}a_{\text{eq}}^{*}}{L^{*}}\text{,}$$

represent characteristic magnitudes of the tensotactic and chemotactic stimuli, respectively. Then, the 1D spatiotemporal evolution of the cell volume fraction, *ϕ*, and flux, *ψ*, in response to interstitial fluid flow, can be solved using Eqs. 31 and 32, together with the constitutive model for the cell stimulus in Eq. 40. We impose no flux boundary conditions at the far field, i.e.,

(Equation 41) $$\frac{\partial \phi }{\partial x}=0\;\text{and}\;\psi =0\;\text{as}\;x\to \pm \infty \text{.}$$

To solve the system of equations given by Eqs. 31, 32, and 40, we use a semi-implicit finite difference scheme; the *x*-derivatives are discretized using a second-order central difference method on a uniform grid spanning the interval [−X,X], where X≫1 is sufficiently large that the far-field boundary conditions given in Eq. 41 have negligible effect on the results. Advancing the system in time is achieved using Euler’s forward method. The above scheme is implemented in MATLAB. The code is available at the following GitHub repository: github.com/yaronbenami/cell_migration.

#### Downstream and upstream migrating populations

An important goal of the present model is to identify parameter regimes in which transitions between upstream and downstream migration occur. While the sign of *ψ* provides an indication of the average direction of cell migration, changes in the proportion of cells traveling upstream and downstream is more accurately given by

(Equation 42) $$\phi^{\text{diff}}=V_{c}\left(\int_{\xi_{x}>0}fd\xi -\int_{\xi_{x}<0}fd\xi \right)\text{.}$$

It is important to note here that, because we used a probabilistic approach to develop our model, we can derive $\phi^{\text{diff}}$ from Eq. 42. This would not have been possible using a conventional multiphase model in which the average macroscopic variables are not explicitly related to microscopic velocity distributions.

Integrating Eq. 2 with respect to ξ for $\xi_{x}>0$ and subtracting the integral of Eq. 2 for $\xi_{x}<0$ we have

(Equation 43) $$\frac{\partial \phi^{\text{diff}}}{\partial t}+\frac{\partial \psi^{\text{diff}}}{\partial x}=\frac{1}{T}\left(\phi \;\tanh\left(\frac{s}{2}\right)-\phi^{\text{diff}}\right)+\frac{1}{{\text{Pe}}_{c}}\frac{\partial^{2}\phi^{\text{diff}}}{\partial x^{2}}\text{,}$$

where

(Equation 44) $$\psi^{\text{diff}}=U_{c}V_{c}\left(\int_{\xi_{x}>0}\xi_{x}fd\xi -\int_{\xi_{x}<0}\xi_{x}fd\xi \right)\text{,}$$

and we note the source (sink) term due to cells changing their migration direction from upstream to downstream (and vice versa). To obtain an equation for $\psi^{\text{diff}}$, we multiply Eq. 2 by $U_{c}V_{c}\xi_{x}$, integrate with respect to ξ for $\xi_{x}>0$, and subtract the integral for $\xi_{x}<0$ to obtain

(Equation 45) $$\frac{\partial \psi^{\text{diff}}}{\partial t}=\frac{U}{T}\left(1-\frac{\tanh\left(s/2\right)}{s}\right)\phi -\frac{\psi^{\text{diff}}}{T}+\frac{1}{{\text{Pe}}_{c}}\frac{\partial^{2}\psi^{\text{diff}}}{\partial x^{2}}\text{.}$$

With ϕ and *s* determined by Eqs. 31, 32, and 40, we can solve Eqs. 43 and 45 to determine $\phi^{\text{diff}}$ and $\psi^{\text{diff}}$.

We define the total difference between downstream- and upstream-migrating cells,

(Equation 46) $$N^{\text{diff}}\left(t\right)=\int_{-\infty }^{\infty }\phi^{\text{diff}}\left(x,t\right)dx\text{,}$$

and use this quantity as a metric to determine whether, at time *t*, there is a dominant tendency for the cells to migrate downstream ($N^{\text{diff}}\left(t\right)>0$) or upstream ($N^{\text{diff}}\left(t\right)<0$). We note that by integrating Eq. 43 with respect to *x* we can derive an ODE for $N^{\text{diff}}$

(Equation 47) $$\frac{dN^{\text{diff}}}{dt}=\frac{R\left(t\right)-N^{\text{diff}}}{T}\text{,}$$

where we have assumed no cell flux as x→±∞ and R(t) is given by

$$R\left(t\right)=\int_{-\infty }^{\infty }\phi \;\tanh\left(\frac{s}{2}\right)dx\text{.}$$

Finally, we define ${\bar{\phi }}_{\text{cr}}\left(t\right)$ as the critical value of $\bar{\phi }$ (the initial volume fraction at x=0, see Eq. 29) for which $N^{\text{diff}}=0$ at time *t*, such that a transition in the overall migration tendency occurs at time *t* when $\phi \left(0,0\right)={\bar{\phi }}_{\text{cr}}$. The experimental significance of ${\bar{\phi }}_{\text{cr}}$ can be summarized as follows. Suppose we want to measure the dominant mode of cell migration after time *T* has elapsed since the seeding of the cells. If the cells are seeded with an initial density ${\bar{\phi }}_{\text{cr}}\left(T\right)$, then equal proportions of cells will be migrating up- and down-stream at time *T*. If the cells are seeded at densities $\bar{\phi }>{\bar{\phi }}_{\text{cr}}\left(T\right)$ then the dominant direction of cell migration at time *T* will be upstream (and vice versa). Therefore, ${\bar{\phi }}_{\text{cr}}$ is a measure that can be used to predict the expected dominant mode of migration at different times during the experiment.

#### Asymptotic analysis of ${\bar{\phi }}_{\text{cr}}$ in the limit of small stimulus

The strengths of the tensotactic and chemotactic stimuli are governed by the nondimensional parameters $\bar{\phi }$ and Da. While the parameters K and M also affect the value of *s*, we will show below that the transition between downstream and upstream migration is dominated by the parameter combinations that yield $s{|}_{x=0}=0$, such that only the ratio K/M affects the value of ${\bar{\phi }}_{\text{cr}}$.

In this section we estimate ${\bar{\phi }}_{\text{cr}}$ in the limiting case for which $\bar{\phi },\;\text{Da}\ll 1$. In this limit, the order-of-magnitude of the stimulus in Eq. 40 scales as

$$s∼O\left({\bar{\phi }}^{2},\text{Da}\bar{\phi }\right)\ll 1\text{.}$$

In what follows we evaluate the asymptotic value of *s* in the limit s≪1. In Eq. 32, in this limit, the cell advective flux, *ψ*, is much smaller than the diffusive flux, $\psi ∼U\bar{\phi }s\ll {\text{Pe}}_{c}^{-1}\bar{\phi }$. Therefore, we may assume that, at leading order, the dynamics of the cell volume fraction are governed by the unsteady diffusion equation

(Equation 48) $$\frac{\partial \phi }{\partial t}\approx \frac{1}{{\text{Pe}}_{c}}\frac{\partial^{2}\phi }{\partial x^{2}}\text{.}$$

The solution of Eq. 48, together with the initial condition

$$\phi \left(x,0\right)=\bar{\phi }\;\exp\;\left(-x^{2}\right)\text{,}$$

and the far-field decay of ϕ, is given by

(Equation 49) $$\phi \left(x,t\right)\approx \frac{\bar{\phi }}{\sqrt{1+4t/{\text{Pe}}_{c}}}\;\exp\;\left(-\frac{x^{2}}{1+4t/{\text{Pe}}_{c}}\right)\text{.}$$

With s≪1, at leading order, Eqs. 43 and 45 yield the following expressions for $\phi^{\text{diff}}$ and $\psi^{\text{diff}}$,

(Equation 50) $$\phi^{\text{diff}}=-T\frac{\partial \psi^{\text{diff}}}{\partial x}+O\left(s\right)$$

and

(Equation 51) $$\psi^{\text{diff}}=\frac{1}{2}U\phi +O\left(s\right)\text{.}$$

Combining Eqs. 50 and 51 we have

(Equation 52) $$\phi^{\text{diff}}=-\frac{1}{2}TU\frac{\partial \phi }{\partial x}+O\left(s\right)\text{.}$$

Since in this limit $\phi^{\text{diff}}$ and $\psi^{\text{diff}}$ are, respectively, proportional to *ϕ* and ∂ϕ/∂x , the unsteady diffusion operator, $L\equiv \partial /\partial t-{\text{Pe}}^{-1}\partial^{2}/\partial x^{2}$ was eliminated from Eqs. 43 and 45 by substituting from Eq. 48.

It can be readily verified from Eq. 52 that $\phi^{\text{diff}}$ is antisymmetric with respect to x=0 (since *ϕ* maintains its symmetry at this limit, see Eq. 49). Thus, the leading order term of $\phi^{\text{diff}}$ does not contribute to the integral in Eq. 46. We conclude that the contribution of the tensotactic and chemotactic stimuli to the integral arises from the O(s) terms localized around x=0, where ∂ϕ/∂x vanishes. Consequently, we can assume that the tendency toward upstream or downstream migration is dominated by the stimulus value at x=0. Substituting Eq. 49 into Eq. 40 we have

(Equation 53) $$s{|}_{x=0}\approx -\frac{K}{\left(1+4t/{\text{Pe}}_{c}\right)}\frac{{\bar{\phi }}^{2}}{{\left(1-\bar{\phi }/\sqrt{1+4t/{\text{Pe}}_{c}}\right)}^{4}}+\frac{M\text{Da}\bar{\phi }}{\sqrt{1+4t/{\text{Pe}}_{c}}}\;\exp\;\left(-\frac{\sqrt{\pi }\text{Da}\bar{\phi }}{2}\right)\text{.}$$

In Eq. 53, we retain terms that are subdominant as $\text{Da},\bar{\phi }\ll 1$. While the higher-order terms are not asymptotically valid (since we did not formally derive the next order correction terms), retaining them in our analysis was useful to capture the qualitative behavior of ${\bar{\phi }}_{\text{cr}}$ for nonsmall Da and $\bar{\phi }$ (see, e.g., the local maximum in ${\bar{\phi }}_{\text{cr}}$ in Fig. 6, which is captured by the asymptotic expression).

In the limit when $\bar{\phi }\ll 1$, $R_{T/C}$, the ratio of the magnitudes of the tensotactic and chemotactic terms in Eq. 53, is proportional to $\bar{\phi }\ll 1$:

$$R_{T/C}=\frac{K\bar{\phi }}{M\text{Da}\sqrt{1+4t/{\text{Pe}}_{c}}}\text{.}$$

We conclude that, for sufficiently small cell volume fractions, there will always be a parameter combination such that $R_{T/C}<1$, i.e., a dominant tendency toward downstream migration. This result is consistent with the experimental findings of Polacheck et al. (5) who observed that downstream migration becomes more dominant as the cell volume fraction decreases (see Fig. 1).

Equating Eq. 53 to zero yields the following transcendental equation for ${\bar{\phi }}_{\text{cr}}$:

(Equation 54) $$\frac{K}{M\text{Da}\sqrt{1+4t/{\text{Pe}}_{c}}}\frac{{\bar{\phi }}_{\text{cr}}\;\exp\;\left(\frac{\sqrt{\pi }\text{Da}{\bar{\phi }}_{\text{cr}}}{2}\right)}{{\left(1-{\bar{\phi }}_{\text{cr}}/\sqrt{1+4t/{\text{Pe}}_{c}}\right)}^{4}}=1\text{,}$$

which depends on the nondimensional parameter groupings, K/M, Da, and $t/{\text{Pe}}_{c}$, representing the relative strength of the tensotactic to chemotactic stimulus, the ratio of chemokine reaction and advection rates, and cell diffusive time, respectively. The dependence of ${\bar{\phi }}_{\text{cr}}$ on *t* means that the critical value of the initial volume fraction for the transition from downstream to upstream migration depends on the time that has elapsed since the cells were seeded. This is because cell diffusion reduces the value of $\phi {|}_{x=0}$ as *t* increases. Consequently, a transition to downstream migration at sufficiently large *t* will always occur, regardless of the value of the initial volume fraction $\bar{\phi }$. Alternatively, by replacing ${\bar{\phi }}_{\text{cr}}\to \bar{\phi }$ and $t\to t_{\text{cr}}$ in Eq. 54, the equation could be interchanged to describe the critical time in which the transition takes place, $t_{\text{cr}}$, as a function of the initial volume fraction $\bar{\phi }$.

## Results and discussion

Table 1 summarizes the nondimensional parameter values used to generate model simulations. We will study the behavior of cells for a range of values of the initial volume fraction, $\bar{\phi }$, and the Damkohler number, Da. These parameters, together with the values of the tensotactic and chemotactic signal strengths, K and M, respectively, govern the magnitude and direction of the stimuli. In this work we keep K and M constant and equal, and vary the values of $\bar{\phi }$ and Da. The cells’ nondimensional velocity, U, and their Peclet number, ${\text{Pe}}_{c}$, are chosen to have physiologically relevant values and are fixed at these default values throughout the paper. We examine two physiologically relevant values of the cells’ relaxation time, T, corresponding to dimensional times of minutes and hours.

**Table 1:** Nondimensional parameter values

Parameter	Value
$\bar{\phi }$	0.01–0.5
Da	0.01–10
U	0.003^a^
${\text{Pe}}_{c}$	300^b^
T	10, 100^c^
K	10
M	10

a Assuming dimensional fluid velocity, $U_{f}^{*}∼1\;\mu m/s$, this gives a dimensional cell speed, $U_{c}^{*}∼10\;\mu m/h$ (5).

b Assuming $U_{f}^{*}∼1\;\mu m/s$ and $L^{*}∼100\;\mu m$, this gives a dimensional cell diffusivity, $D_{c}^{*}∼1000\;\mu m^{2}/h$ (33).

c See discussion after Eq. 32 et seq.

**Figure 3:**
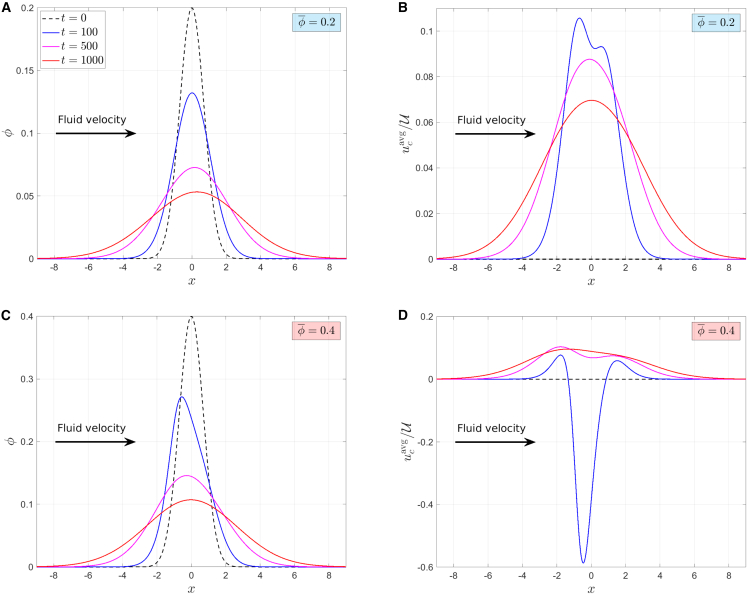
The spatiotemporal evolution of the cell volume fraction (*A* and *C*) and scaled average cell velocity (*B* and *D*) starting from a Gaussian *x*-distribution of cells at t=0, with $\bar{\phi }=0.2$ (*A* and *B*) and $\bar{\phi }=0.4$ (*C* and *D*). The solutions are plotted at times t=0 (*black dashed line*), t=100 (*blue*), t=500 (*magenta*), and t=1000 (*red*). Parameter values: Da=0.5, T=10; other parameters are fixed at the values listed in Table 1. The arrows indicate the direction of the fluid velocity (i.e., the downstream direction). Positive and negative velocities (in *B* and *D*) represent average cell velocities that are oriented downstream and upstream, respectively. To see this figure in color, go online.

Fig. 3 illustrates the spatiotemporal evolution of the cell layer in response to the flow-induced chemotactic and tensotactic stimuli. We define the macroscopic average cell velocity as

(Equation 55) $$u_{c}^{\text{avg}}=\frac{\psi }{\phi }\text{,}$$

and plot the *x*-distributions of the scaled average velocity, $u_{c}^{\text{avg}}/U$ (macroscopic cell velocity normalized by the individual cell velocity), and the cell volume fraction, *ϕ*, at times t=0,100,500,1000. For the specific case of Da=0.5 and T=10, we consider two initial cell volume fractions, $\bar{\phi }$, which induce different migration behaviors: in Fig. 3, *A* and *B*, $\bar{\phi }=0.2$, leading to dominant downstream migration at all times, as can be seen from the average cell velocity profiles in Fig. 3 *B*, which are always nonnegative. By contrast, in Fig. 3, *C* and *D*, $\bar{\phi }=0.4$, leading to upstream migration at early times, and a transition to dominant downstream migration at later times (notice the dominant negative cell velocity at early times in Fig. 3 *D*, as indicated by the t=100 curve, which changes to all-positive velocity profiles at t>500).

**Figure 4:**
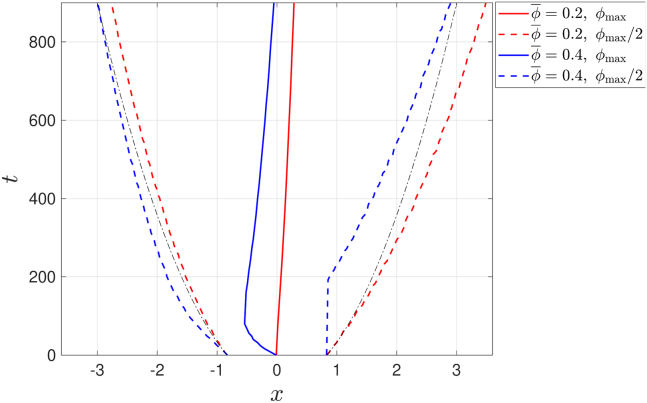
Different regions of the cell layer travel at different velocities. The change in the spatial position, *x*, of $\phi_{\max}$ (*solid lines*) and $\phi_{\max}/2$ (*dashed lines*) as a function of time, for the two cases presented in Fig. 3, $\bar{\phi }=0.2$ (*red lines*) and $\bar{\phi }=0.4$ (*blue lines*). For comparison, the dashed-dotted black lines show the purely diffusive evolution of the spatial location of $\phi_{\max}/2$. To see this figure in color, go online.

To complete the picture of the different velocities in which different regions of the cell layer migrate, Fig. 4 shows how the spatial position, *x*, at which the volume fraction attains its maximal value, $\phi_{\max}$, changes over time *t* (solid lines). Also shown are the trajectories of the two spatial locations at which the volume fraction attains its half-maximal value (dashed lines). For purely diffusive (PD) motion (i.e., without directed migration, ψ=0), the cell flux is PD (i.e., in the direction of −∂ϕ/∂x). The PD trajectory, $x_{\text{PD}}$, for which $\phi =\phi_{\max}/2$, is given by

(Equation 56) $$x_{\text{PD}}\left(t;\phi =\phi_{\max}/2\right)=\pm \ln\left(2\right)\sqrt{1+4t/{\text{Pe}}_{c}}$$

and is included in Fig. 4 for reference. Naturally, in the PD case the location of the maximal value does not change over time, $x_{\text{PD}}\left(t;\phi =\phi_{\max}\right)=0$.

Comparing the PD trajectories (*black dashed-dotted lines*) and the red trajectories ($\bar{\phi }=0.2$) in Fig. 4, we note that both trajectories exhibit the same qualitative behavior, except for a small, right (downstream) shift of the red curves due to the stimulus-induced directed motion. This means that, when $\bar{\phi }=0.2\text{,}$ we have a dominant cell diffusion, where the locations of $\phi =\phi_{\max}/2$ predominantly travel in the direction opposite to the volume fraction gradient. This diffusive movement is superimposed on a small shift of the cell layer to the right (downstream) due to the chemotaxis-induced positive velocity. For low cell volume fractions the effect of the stimulus-induced migration is small due to the small average cell velocity (up to 10% of the individual cell speed in the case of $\bar{\phi }=0.2$, see Fig. 3 *B*). This is because, although chemotaxis dominates tensotaxis, the chemotactic signal is rather weak at low volume fractions. While the cell layer in Fig. 3 *A* seems to maintain its symmetry with respect to the location of the maximum volume fraction, it is possible to detect a small amount of symmetry breaking in the cell velocity field due to the nonlinearity of the tensotactic and chemotactic stimuli, which attain their maximum values at slightly different *x*-locations.

At a larger initial cell volume fraction, in Fig. 3 *C* we can notice that the cell layer is skewed to the left at early times due to the large negative cell velocity at the center of the cell layer (*solid blue line* in Fig. 4). This is because the tensotactic signal is dominant in the region where the volume fraction is large. At early times we also notice that the stimulus-directed velocity and diffusive velocity are opposite at the downstream part of the cell layer, while they reinforce each other to generate a large upstream velocity at the upstream part of the cell layer (compare the *blue dashed curves* and PD curves in Fig. 4). At later times, we observe a reduction in the cell volume fraction due to the action of cell diffusion, which consequently leads to a transition to dominant downstream migration (notice the change to positive velocity in Fig. 3 *D* and in the *solid blue line* in Fig. 4 when t≳100).

**Figure 5:**
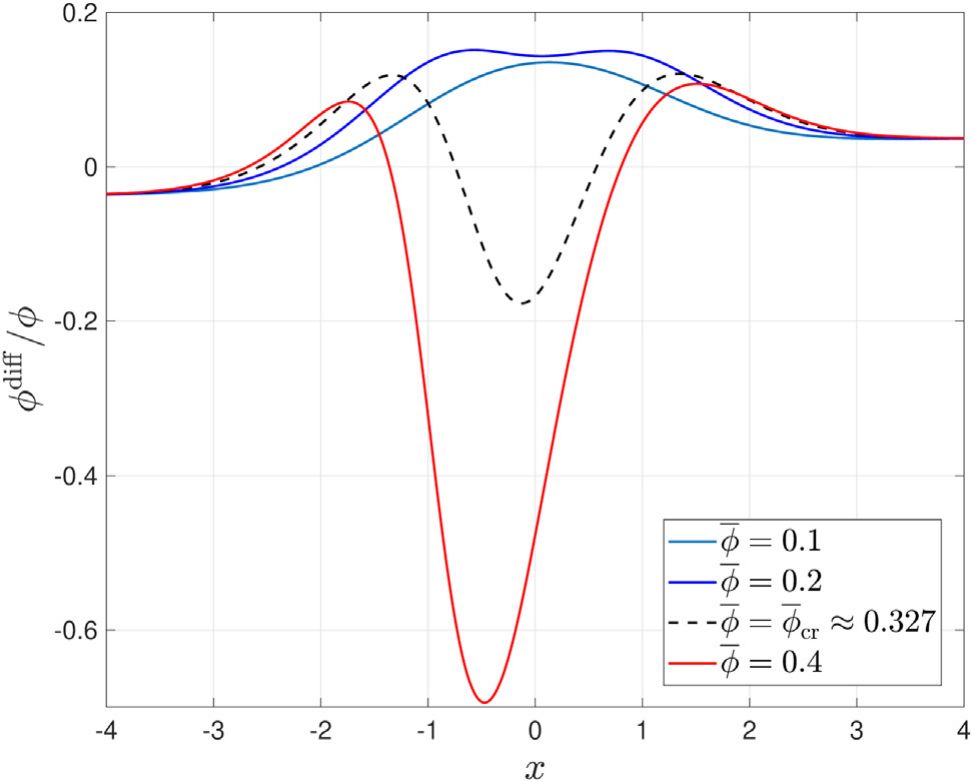
Series of plots showing how, at a fixed time point t=100, the proportion of cells moving upstream and downstream changes with *x* for different values of the initial volume fraction, $\bar{\phi }$. The black dashed line represents the critical volume fraction, ${\bar{\phi }}_{\text{cr}}$, at which the dominant mode of migration switches between downstream and upstream. Parameter values: Da=0.5, T=10; other parameters use the values listed in Table 1. To see this figure in color, go online.

To better illustrate the change in the dominant mode of migration as the cell volume fraction changes, the results presented in Fig. 5 show the spatial variation in the proportion of cells traveling downstream and upstream at time t=100 when Da=0.5 and the initial volume fraction varies. For small $\bar{\phi }$, most cells travel downstream. As $\bar{\phi }$ increases, more cells travel downstream (compare the change between $\bar{\phi }=0.1$ and 0.2 in Fig. 5) due to the increased production of chemokine; however, the maximum number of cells traveling downstream is no longer in the center of the cell layer due to the increased tensotactic stimulus in the region where the cell volume fraction is maximal. As $\bar{\phi }$ increases further, different migration directions dominate in different regions of the cell layer. On the one hand, the strong tensotactic stimulus in the center of the cell layer, where the cell volume fraction is maximal, leads to upstream migration in this region; on the other hand, cells in the edges, where the volume fraction is smaller, continue to migrate downstream. At the critical value, ${\bar{\phi }}_{\text{cr}}$ (*black dashed line* in Fig. 5), there is a balance between the proportion of upstream-migrating cells in the bulk of the cell layer and the proportion of downstream-migrating cells at the edges of the cell cluster. As $\bar{\phi }$ increases beyond ${\bar{\phi }}_{\text{cr}}$ more cells migrate upstream. Due to the strong nonlinearity of the tensotactic stimulus, small deviations of $\bar{\phi }$ above ${\bar{\phi }}_{\text{cr}}$ amplify the tendency for upstream migration. Consequently, the *x*-position where $\phi^{\text{diff}}$ attains its minimal value (in the region of dominant tensotaxis) moves to the left as $\bar{\phi }$ increases because of the large cell flux in the negative *x* direction, which shifts the location of the maximum value of ϕ.

Sufficiently far from the bulk of the cell layer, where the stimuli are very weak and cell diffusion dominates, all curves of $\phi^{\text{diff}}$ in Fig. 5 collapse onto a single curve. In these regions cells tend to diffuse in the direction of decreasing cell density.

**Figure 6:**
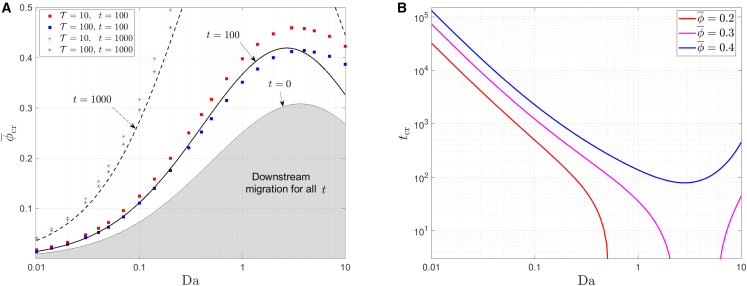
The critical conditions for transition between dominant downstream and upstream migration in the parameter space of Da, $\bar{\phi }$, and *t*. (*A*) The critical cell volume fraction, ${\bar{\phi }}_{\text{cr}}$, for transition as a function of Da. Comparison between the asymptotic expression (Eq. 54) at times $t_{\text{short}}=100$ (*solid line*) and $t_{\text{long}}=1000$ (*dashed line*) and numerical simulation results at the respective times (*square* and *star symbols*, respectively). Two values of cell relaxation times, T=10 (*red symbols*) and T=100 (*blue symbols*) were simulated. The shaded gray area indicates the region in parameter space in which upstream migration does not take place for any *t*. (*B*) The transition time, $t_{\text{cr}}$, as a function of Da for different values of $\bar{\phi }$, as predicted by the asymptotic model. To see this figure in color, go online.

Having observed transitions in the favorable migration direction as the system parameters vary, it is useful to delineate the parameter regimes in which these transitions occur. For that purpose, the results presented in Fig. 6 *A* show how ${\bar{\phi }}_{\text{cr}}$ changes as the Damkohler number, Da, is varied for two fixed values of the cell relaxation time, T, and two values of the time that has elapsed since the initial condition; all other parameters are held fixed at their default values (see Table 1). The characteristic time scale is given by $L^{*}/U_{f}^{*}∼100\;s$; we used values of T corresponding to dimensional relaxation times of several minutes (T=10, $\tau^{*}∼17\;\min$, *red symbols*) and a few hours (T=100, $\tau^{*}∼3\;h$, *blue symbols*) corresponding to physiologically relevant relaxation times (30). For the time at which data were collected (i.e., the elapsed time since the start of the experiment), we used dimensional times of several hours ($t_{\text{short}}=100$, *square symbols*) and approximately one day ($t_{\text{long}}=1000$, *star symbols*) to study cell behavior on time scales that are either much smaller or similar in magnitude to the time scale for cell migration, respectively. For each parameter combination we used the MATLAB function fzero, and our numerical scheme, to determine the value of $\bar{\phi }$ for which $N^{\text{diff}}=0$ at the simulated time (either $t_{\text{short}}$ or $t_{\text{long}}$).

Fig. 6*A* shows that ${\bar{\phi }}_{\text{cr}}$ increases as the duration of time for which data are collected increases. This can be rationalized by noting that, as *t* increases, the cell volume fraction decreases due to cell diffusion; thus, the tendency for upstream-oriented tensotactic migration decreases. Therefore, if the time at which we measure the mode of migration increases, then the initial volume fraction, $\bar{\phi }$, must also be increased so that a mode transition (equal proportion of cells migrating down- and up-stream) occurs at this time. The gray region in Fig. 6 *A* indicates the parameter region in which downstream migration prevails for all *t*. This region is delineated by the critical curve, ${\bar{\phi }}_{\text{cr}}\left(\text{Da};t=0\right)$, on which a “transition” between upstream and downstream migration occurs at time t=0. Consequently, for each value of Da, if $\bar{\phi }$ is outside this gray region, upstream migration will dominate at early times, and a transition to downstream migration will occur at some later time. In more detail, a parameter combination in the area delineated by the two curves in Fig. 6 *A*, ${\bar{\phi }}_{\text{cr}}\left(\text{Da};t=t_{1}\right)$ and ${\bar{\phi }}_{\text{cr}}\left(\text{Da};t=t_{2}\right)$, will undergo a transition between upstream and downstream migration at some time in the interval $t\in \left(t_{1},t_{2}\right)$. Due to the action of cell diffusion, the cell volume fraction reduces over time and favors downstream migration at later times. Due to this mechanism, we expect that, at sufficiently large times, downstream migration will always prevail. However, depending on the values of the parameters, these times could be extremely long and may not be physiologically relevant. For example, the dimensional long time we used is equivalent to $t_{\text{long}}^{*}∼1\;\text{day}$. This is about the maximum time scale on which the current model is applicable since, at longer time scales, processes such as cell proliferation and death may no longer be negligible. To further illustrate the effect of the time that has elapsed since the beginning of the experiment on the migration behavior, Fig. 6 *B* shows the transition time, $t_{\text{cr}}$, as a function of Da for a range of values of $\bar{\phi }$. In accordance with the gray region in Fig. 6 *A*, for sufficiently small values of $\bar{\phi }$, there is a range of values of Da for which physically realistic values of $t_{\text{cr}}$ do not exist (i.e., $t_{\text{cr}}<0$ in this region) and, thus, downstream migration prevails at all times. As expected, $t_{\text{cr}}$ increases as $\bar{\phi }$ increases, reflecting the increase in tensotactic stimulus with the cell volume fraction increases.

While the time that has elapsed since the initial state may affect the value of ${\bar{\phi }}_{\text{cr}}$ dramatically, Fig. 6 *A* shows that varying the cell relaxation time by a factor of 10 (while remaining in the physiologically relevant regime) does not significantly alter the critical value of ${\bar{\phi }}_{\text{cr}}$ (compare the *red* and *blue symbols* in Fig. 6 *A*). The modest increase in ${\bar{\phi }}_{\text{cr}}$ for smaller relaxation times can be attributed to a more rapid reaction of the cells to changes in the dominant external stimulus from tensotaxis-dominated stimulus at early times to chemotaxis-dominated stimulus at later time. This, in turn, causes the transition to occur at earlier times.

The black lines in Fig. 6 *A* correspond to the asymptotic behavior of ${\bar{\phi }}_{\text{cr}}$ as Da≪1 and $\bar{\phi }\ll 1$, given by Eq. 54, for t=100 (*solid line*) and t=1000 (*dashed line*). We note that the asymptotic model is in excellent agreement with the numerical results when $\bar{\phi },\text{Da}\ll 1$. We note that it also replicates the general trend for larger values of $\bar{\phi }$ and Da. As expected, the agreement improves when the cell relaxation time increases or the elapsed time decreases. This is because larger relaxation times and smaller elapsed times mean less skewness of the cell distribution with respect to their *x*-symmetric initial distribution, such that the assumptions on which the asymptotic model is based (see Eq. 48 et seq*.*) are better fulfilled.

In the limit of $\bar{\phi }\ll 1$ it can be readily seen from the asymptotic expression in Eq. 54 that, when K/M decreases and Da increases (for example, by reducing the fluid velocity), the critical volume fraction for transition from downstream to upstream migration increases. Indeed, in (5) smaller fluid velocities were found to reduce the tendency for upstream migration (see Fig. 1). Based on our model, we attribute this behavior to the impact that a reduction in the fluid velocity has on the mechanisms that inhibit upstream migration and promote downstream migration: 1) the fluid cell drag force decreases, which leads to a smaller transcellular pressure gradient and a smaller tensotactic cue, 2) the ratio of reaction to advection increases, which leads to larger chemokine gradients and a larger downstream-oriented chemotactic signal. For large values of the Damkohler number (Da≳3), we note a qualitative change in behavior where further increases in Da lead to a reduction in ${\bar{\phi }}_{\text{cr}}$ (i.e., a reduced tendency to migrate downstream). This is due to increased chemokine consumption by the cells as the chemokine concentration increases, which diminishes the chemotactic gradients in the downstream region of the cell layer. This nonmonotonic behavior of the critical conditions with respect to the Damkohler number is also shown in Fig. 6 *B*, where the transition time initially decreases with Da (corresponding to the aforementioned increase in the chemotactic stimulus), but starts to increase at O(1) values of Da.

## Conclusion

The goal of this study was to use mathematical modeling to study cell migration in response to flow-induced mechanical and chemical stimuli. We developed a hybrid probabilistic continuum model for a two-phase mixture of fluid and cells. We started from a mesoscopic kinetic description for the cell probability density function, forced by a stimulus-dependent TPF biasing the cell velocity probability. Then, we used velocity-space averaging to formulate a system of continuum equations that describe how the cells’ spatial distribution evolves over time at the macroscopic level in response to the mechanochemical signal. The use of a kinetic description as a starting point to derive continuum models for cell migration has been widely used (e.g., (21,24,36,37,38)); in addition, there are several hybrid models of cell chemotaxis (e.g., (39,40,41)), in which the transport of a chemoattractant is described by a macroscopic equation, with source/sink terms that depend on the density of cells, while the cells are described at the kinetic level. However, our model also accounts for tensotactic migration of cells. Since tensotactic migration depends on the pressure distribution, this necessitates the formulation of equations for fluid flow, which are coupled to cell motion via the incompressibility of the mixture and the dependence of the permeability on the cell volume fraction. To the best of our knowledge, coupling of this kind has not been carried out before.

Motivated by the experimental results by Polacheck et al. (5), we focused on studying the migration of a 1D cell layer in an infinite channel subject to a fluid flow. Contrary to purely continuum-based models, our probabilistic approach enabled us to determine how the proportion of cells traveling upstream and downstream at a given spatial location evolves over time, and to determine the critical conditions at which transitions in the dominant mode of migration occur.

Through a combination of numerical simulation of the 1D model and asymptotic analysis, we delineated the locus of transitions in the two-parameter plane defined by the initial cell volume fraction, $\bar{\phi }$, and the Damkohler number, Da, the latter parameter representing the ratio of chemokine secretion to advection rates. In agreement with the experimental observations in (5) (see Fig. 1), the current model predicts downstream-oriented chemotactic migration at low cell volume fractions, and upstream-oriented tensotactic migration at larger volume fractions. This effect can be understood by the increase in the transcellular pressure gradient and consequent tensotactic stimulus when the cell volume fraction increases.

In the experiments by Polacheck et al. (5), the distribution of cell velocity was only measured at a single time point. By contrast, our model predicts that the time at which experimental data are collected has an important effect on the observed dominant mode of migration. We identified a region of the parameter space in which the chemotactic stimulus dominates the tensotactic stimulus for all *t* and, thus, downstream migration prevails for the duration of the experiment. By contrast, outside this region of parameter space, upstream migration prevails at the beginning of the experiment when the cells are localized, and a transition to downstream migration occurs at later times, due to the effect of cell diffusion, which causes the distribution of cells to become more dispersed over time. However, this initial dominant-upstream-migration transient can persist up to very long times ($t>1000\to t^{*}>1\;\text{day}$ for some regions of parameter space). This means that it will be clearly visible in cell migration experiments (e.g., in (5) measurements were taken after 24 h). This phenomenon may indicate the need to measure the cell velocities at different time points when conducting cell migration experiments.

We additionally showed that an increase in Da tends to increase the importance of chemotactic migration due to enhanced chemokine secretion by the cells. However, our model predicts that there is an optimal value of Da∼O(1), which maximizes the chemotactic signal; as Da increases above this local maximum, chemokine degradation increases, leading to smaller chemotactic gradients in the downstream region of the cell layer. Here, we mention that the current results were obtained in the purely advective limit of the chemokine propagation, i.e., neglecting diffusive effects. It is expected that including chemokine diffusion and boundary interactions (e.g., no flux) will result in a more complicated behavior.

Applying asymptotic analysis in the limit of ϕ,Da≪1, we obtained an explicit formula for the critical conditions in terms of the system parameters. The asymptotic expression showed excellent agreement with the numerical results in the limit of ϕ,Da≪1, while it was also able to capture the general trend at larger values of ϕ and Da, including the local maximum observed in the numerical results.

An important feature of the current model is the use of a permeability function that depends on the cell volume fraction, ensuring that tensotactic stimulus increases as the cell volume fraction increases. While in this work we used the isotropic Carman-Kozney permeability, in future work it would be interesting to examine alternative permeability models that account for the anisotropic, fibrous nature of the ECM (e.g., (42)).

While not considered in this paper, the kinetic model given by Eq. 2 also allows one to calculate the microscopic cell velocity probability distribution, f(x,t,ξ). This would need to be carried out via a hybrid model where the numerical solution of *f*, obtained from the kinetic model in Eq. 2, is averaged to produce the macroscopic cell volume fraction. This, in turn, is coupled to the fluid motion and chemoattractant transport, affecting the macroscopic stimulus profile; the stimulus is then fed back to the transition probability, *F*, in the kinetic equation. In future work, such a hybrid model could be used to evaluate the cell velocity distribution, which could be compared with experimental measurements of the distribution of cellular velocities. In this way, it should be possible to refine the functional form of the TPF, F(x,t,ξ), to achieve better agreement with the experimental results.

Finally, the model developed in this paper constitutes a novel framework to study cell migration in a dynamic fluid environment. One example for such cell migration is the movement of tumor cells toward plasma-depleting blood vessels which can lead to either vessel collapse (43) or intravasation (2). Here, the interaction of the cells with the vessel walls may affect the flux of interstitial fluid depleted by the vessel, thus coupling the extravascular cell migration to intravascular blood flow. This phenomenon has significant implications for tumor blood flow, progression, and therapy (44,45), and thus represents a natural topic for future work.

## Author contributions

Y.B.-A. designed and performed the research and wrote the manuscript. J.M.P.-F., P.K.M. and H.M.B. designed the research and wrote the manuscript.
